# A case of Anti-Hu and Anti-Zic4 paraneoplastic cerebellar degeneration in a patient with stage IV adenocarcinoma of Müllerian origin

**DOI:** 10.1016/j.gore.2025.101804

**Published:** 2025-07-09

**Authors:** Saad Rashid, Suneha Pocha, Nada Saleh, Erin Ozminkowski, Vijayta Geeta Bansal, Vibhav Bansal

**Affiliations:** a Internal Medicine Program, Mercyhealth Graduate Medical Education Consortium, Rockford, IL, USA; b Department of Neurology, Great Lakes Institute of Neurology, Libertyville, IL, USA; c Department of Neurology, Mercyhealth, Rockford, IL, USA

**Keywords:** Paraneoplastic cerebellar degeneration, Ovarian cancer, anti-Hu, Corticosteroids, Malignancy

## Abstract

**Introduction:**

Paraneoplastic cerebellar degeneration (PCD) is a rare neurological complication of malignancies, resulting from autoimmune responses targeting antigens shared by neurons and tumors. It is characterized by progressive cerebellar dysfunction, including ataxia and dysarthria, and is associated with anti-onconeuronal antibodies such as Anti-Yo and Anti-Hu. While most cases of Anti-Hu-associated PCD occur in patients with small cell lung cancer, their association with gynecologic malignancies is exceedingly rare. This is the first reported case in the literature of a patient with adenocarcinoma of Müllerian origin complicated by Anti-Hu PCD.

**Case:**

We present the case of a 73-year-old female with stage IV adenocarcinoma of Müllerian origin who presented with worsening ataxia and dysarthria after undergoing outpatient chemoimmunotherapy to treat her gynecologic malignancy. CT and MRI Brain imaging revealed nonspecific findings, but cerebrospinal fluid (CSF) analysis revealed the presence of positive anti-Hu and anti-Zic4 antibodies, consistent with a diagnosis of PCD. Despite treatment with high-dose intravenous corticosteroids and plasmapheresis in the acute setting, the patient displayed minimal improvement and continued to experience persistent neurological impairment.

**Conclusion:**

Despite advancements in diagnostic criteria and the availability of multiple types of therapies, outcomes for PCD remain poor. This is especially notable for cases associated with Anti-Hu antibodies. This case highlights the importance of considering PCD in the differential diagnosis of patients with malignancies and unexplained neurological symptoms. Furthermore, it highlights the critical need for continued research to refine diagnostic methods and develop more effective, evidence-based treatment strategies to improve outcomes.

## Highlights

• Paraneoplastic cerebellar degeneration (PCD) presents as progressive cerebellar dysfunction.
• Diagnosis of PCD includes physical examination, imaging, cerebrospinal fluid analysis, and testing for onconeural antibodies.
• Several onconeural antibodies are associated with PCD, including anti-Yo, anti-Tr, anti-Ri, and anti-Hu.
• Treatment modalities include primary tumor resection, corticosteroids, IVIG, and plasmapheresis.
• PCD should be considered in patients with known malignancy when neurologic symptoms arise.

## 1 Introduction

Paraneoplastic neurological syndromes (PNSs) represent a rare group of immune-mediated disorders that develop in fewer than 1 % of cancer patients. These syndromes can be profoundly debilitating due to their severe neurological impairments (Braik et al., 2010). They are commonly associated with malignancies such as breast cancer, small cell lung cancer, Hodgkin lymphoma, and gynecologic cancers (Cui et al., 2017). PNSs typically arise due to tumor-induced immune responses targeting neuronal antigens, impacting the central, peripheral, and autonomic nervous systems, as well as the neuromuscular junction. Diagnosing PNSs is challenging and requires exclusion of direct nervous system involvement by the malignancy (such as via brain metastasis or carcinomatous meningitis) and indirect effects, such as by coagulopathy and treatment-related toxicity (Honnorat and Antoine, 2007). Notable examples of PNSs include Lambert-Eaton myasthenic syndrome, anti-NMDA receptor encephalitis, and paraneoplastic cerebellar degeneration (PCD).

Paraneoplastic cerebellar degeneration (PCD) constitutes a rare and heterogeneous group of neurological disorders resulting from tumor-induced autoimmunity against cerebellar antigens (Venkatraman and Opal, 2016). It is characterized by an acute or subacute onset of pan-cerebellar dysfunction, including gait ataxia, dysarthria, diplopia, nystagmus, and limb asymmetry (Anderson et al., 1988). Several specific anti-onconeural antibodies found in the serum and cerebrospinal fluid have been linked to PCD. Among these, Anti-Yo antibodies are the most frequently identified, particularly in cases associated with breast and gynecological cancers. Other antibodies, such as Anti-Ri and Anti-Hu, are much rarer.

We present the first reported case of paraneoplastic cerebellar degeneration with Anti-Hu and Anti-Zic4 antibodies in a 73-year-old patient with stage IV adenocarcinoma of Müllerian origin. This case highlights the diagnostic challenges, treatment strategies, and clinical significance of paraneoplastic cerebellar degeneration. Additionally, we contextualize the case within the existing literature.

## 2 Case presentation

A 73-year-old female with a past medical history of essential hypertension and stage 3 chronic kidney disease was initially diagnosed with a high-grade gynecological malignancy whilst undergoing evaluation for worsening back pain. CT Abdomen/Pelvis without Contrast imaging revealed extensive bilateral retroperitoneal lymphadenopathy. Left retroperitoneal core lymph node biopsy showed poorly differentiated carcinoma, positive for PAX8, ER, WT1, p16, p53, and FR-alpha, consistent with Müllerian origin. Pathology review further suggested uterine papillary serous carcinoma. Positron Emission Tomography (PET) imaging revealed extensive bilateral supraclavicular, mediastinal, intrathoracic, retroperitoneal, and right external iliac adenopathy, without evidence of malignancy on upper and lower endoscopy or mammography. No definitive primary mass was identified on imaging. Laboratory studies were notable for markedly elevated CA-125 levels of 6650. The patient established care with Gynecology Oncology and Medical Oncology in the outpatient setting, with a working diagnosis of stage IV adenocarcinoma of Müllerian origin. Given concern for an endometrial primary, Medical Oncology initiated systemic therapy with a regimen of paclitaxel, carboplatin, and pembrolizumab. Repeat PET imaging after three cycles showed improvement in lymphadenopathy. The plan was to complete six total cycles of therapy, followed by re-imaging. Gynecology Oncology planned for surgical excision, including total abdominal hysterectomy, if disease progression was noted on follow-up imaging.

During the fourth cycle of this systemic therapy, the patient presented to the Emergency Department as a transfer from an outside hospital for evaluation of worsening aphasia and ataxia, leaving the patient unable to ambulate independently. Her symptoms had begun approximately one month ago, with the onset of slurred speech and unsteady gait. This had culminated in a mechanical fall at home in her bathroom. During the fall, she sustained trauma to her left hip and face, prompting an initial visit to the emergency department for evaluation of an acute stroke.

During the prior inpatient admission, Computed Tomography (CT) Head without Contrast and Magnetic Resonance Imaging (MRI) Brain without Contrast imaging showed no abnormalities. CT Angiography Head and Neck revealed high-grade stenosis of the left external carotid artery. The patient was discharged home, with her symptoms attributed to polypharmacy. The patient was taking prescribed opioids and muscle relaxants to manage chronic back and cancer-related pain. During her scheduled outpatient Medical Oncology follow-up visit, she was recommended to re-present to the Emergency Department due to the continuation of her symptoms, alongside worsening headache.

Physical examination at this time revealed dysarthria, bilateral horizontal and vertical nystagmus, bilateral dysmetria, and marked ataxia on finger-to-nose and finger-to-shin testing, without any associated weakness. Initial vital signs demonstrated normotensive blood pressure, without any fever, hypoxia, or tachycardia.

General Neurology was consulted during this admission. Given the constellation of bilateral cerebellar symptoms, the differential diagnosis included leptomeningeal carcinomatosis and paraneoplastic syndrome. Repeat MRI Brain with and without intravenous contrast was negative for acute processes, including metastasis or stroke. A lumbar puncture was performed, revealing elevated cerebrospinal fluid (CSF) protein of 98. CSF electrophoresis revealed increased IgG and myelin basic protein levels. Cytology was negative for malignant cells. Table 1 provides a summary of comprehensive CSF testing results. A serum paraneoplastic antibody panel tested positive for Anti-Hu antibodies. CSF autoimmune testing detected positive Anti-Hu and Anti-Zic4 antibodies, confirming the diagnosis of paraneoplastic cerebellar degeneration.

**Table 1:** Select Cerebrospinal Fluid Analysis Results.

**CSF Parameter**	**Lab Value**	**Normal Range**
Xanthochromia	Negative	Negative
WBC	107/mm^3^	0–5/mm^3^
Mononuclear Cells (%)	98 %	≤70 %
RBC	6/mm^3^	0/mm^3^
Glucose	58 mg/dL	40–70 mg/dL
Total Protein	98 mg/dL	15 – 45 mg/dL
Anti-Hu Antibody	Positive	Negative
Anti-Ri Antibody	Negative	Negative
Anti-Yo Antibody	Negative	Negative
Zic4 Antibody	Positive	Negative
Myelin Basic Protein	10.9 ng/mL	0.0 – 5.6 ng/mL
ACE CSF	Negative	Negative
West Nile CSF Antibody	Negative	Negative
Meningitis Panel	Negative	Negative
Gamma Globulin	27.9 %	3.1 % − 9.1 %
Oligoclonal Bands	3 bands	<4

Treatment for PCD was initiated with five days of high-dose intravenous methylprednisolone (1 g/day) and five sessions of plasmapheresis. The patient demonstrated minimal improvement but experienced no progression of her symptoms. Upon completion of corticosteroid therapy and plasmapheresis, the patient was discharged to a skilled nursing facility with scheduled outpatient follow-ups with Medical Oncology, Gynecology Oncology, and Neurology. During follow-up with Neurology, the patient’s cerebellar symptoms persisted, and she remained unable to ambulate independently.

## 3 Discussion

Paraneoplastic neurological syndromes (PNSs) are rare immune-mediated complications arising from malignancy-induced autoimmune responses against neuronal antigens. Paraneoplastic cerebellar degeneration (PCD), a type of PNS, manifests as progressive cerebellar dysfunction due to tumor-triggered neuronal damage. Symptoms of cerebellar dysfunction include gait ataxia, diplopia, nystagmus, dysmetria, and dysarthria, with gait ataxia often presenting as the primary or sole initial feature. These symptoms typically progress over weeks to months and can lead to significant functional impairment (Graus et al., 2021).

The pathogenesis of PCD remains incompletely understood but is thought to involve antibody and T-cell-mediated immune responses against shared epitopes expressed in the nervous system and the primary tumor (Bhat and Steinman, 2009). Onconeural antigens, ectopic neuronal proteins expressed by tumor cells, trigger immune system activation and lead to neuronal destruction (Deac et al., 2020).

PCD can occur at any stage of cancer, with up to 30 % of symptomatic cases emerging during cancer remission (Cui et al., 2017). Neurological symptoms often precede the diagnosis of the underlying malignancy, though they may also arise during tumor recurrence or progression. In our case, the patient developed PCD after undergoing chemoimmunotherapy for a known malignancy.

The diagnosis of PCD requires a thorough evaluation, including physical examination, imaging studies, CSF analysis, and serological testing for onconeural antibodies. In 2019, the PNS-Care Panel revised the diagnostic guidelines to refine clinical decision-making strategies in patients with PNSs. Specifically for PCD, the PNS-Care Panel emphasized that truncal and limb involvement must be seen during the disease course to meet the diagnosis (Graus et al., 2021).

Imaging modalities, such as CT and MRI, are critical for ruling out structural abnormalities, such as stroke, tumor, or metastasis. In PCD, imaging is often non-diagnostic. However, patients with advanced cerebellar degeneration may display cerebellar atrophy observable on MRI imaging (Graus et al., 2021). Serum and CSF testing for onconeural antibodies is pivotal, as antibodies linked to CNS involvement are more concentrated in the CSF and may be undetectable in the serum. In this case, both serum and CSF antibody titers were revealing for Anti-Hu antibodies.

PCD is associated with many specific onconeural antibodies, including anti-Yo, anti-PCA2, anti-Hu, anti-Tr, anti-Ri, anti-mGluR1, anti-Zic4, and anti-Ma, among others (Deac et al., 2020). However, up to 40 % of patients with PCD lack detectable antibodies, presenting further diagnostic challenges (Dalmau et al., 2007). Anti-Zic4 antibodies are directed against the zinc-finger domain of the intracellular transcription factor Zic4, which plays a crucial role in CNS development (Bataller et al., 2004). PCD is the most common syndrome in patients with anti-Zic4 antibodies, which frequently co-occur with other onconeural antibodies, such as anti-Hu. Greater than 90 % of cases of anti-Zic4-associated PCD have been reported in patients with small cell lung cancer (Sabater et al., 2008). Our case represents the first reported instance of Anti-Hu-associated PCD in a patient with adenocarcinoma of Müllerian origin and one of very few cases involving Anti-Zic4 antibodies in this malignancy (Kerasnoudis et al., 2011).

The prognosis for PCD patients is primarily influenced by the severity of their neurological syndrome rather than the underlying malignancy. Shams’ili et al. conducted a retrospective analysis of 50 PCD patients, identifying marked variability in outcomes among those with different antineuronal antibody subtypes. Among patients with anti-Hu antibodies, only 25 % (4/16 patients) remained ambulatory after diagnosis, with a mean survival of 7 months from the diagnosis (Shams'ili et al., 2003). In contrast, patients with anti-Ri antibodies demonstrated improved functional outcomes, with 83 % (5/6 patients) maintaining ambulatory status and a median survival of > 69 months (Shams'ili et al., 2003). These findings highlight the variability in prognosis in PCD based on antibody subtypes, with Anti-Hu antibodies carrying worse outcomes. In our case, the patient continued to experience substantial cerebellar dysfunction despite therapeutic intervention.

Treatment of PCD centers on identifying and treating the underlying malignancy. Additional therapeutic approaches aimed at mitigating neurological symptoms include plasma exchange, intravenous immunoglobulin (IVIG), corticosteroids, and immunosuppressive agents (such as cyclophosphamide) (Kannoth, 2012). Due to the rarity of PCD and limited published cases, standardized treatment protocols have not been established (Shams'ili et al., 2003). Plasma exchange offers nonspecific removal of antibodies and immune complexes, alongside inflammatory mediators such as cytokines. Previous isolated cases of PCD have reported benefit with plasma exchange (Greenlee, 2013). The use of IVIG remains speculative, with proposed mechanisms including reduction of T-cell proliferation and suppression of pro-inflammatory cytokines (Gelfand, 2012). Corticosteroid regimens typically involve high-dose intravenous methylprednisolone (1000 mg daily for three to five days), often followed by an oral prednisone taper (Greenlee, 2013). Immunosuppressive agents, such as cyclophosphamide, primarily impact T-cell activity but lack specific effects on circulating antibodies in the serum and CSF. In our case, the patient received high-dose corticosteroids and plasma exchange during her inpatient stay.

Table 2 highlights many of the reported cases of PCD in the literature, including patients’ age, gender, primary tumor, identified antibodies, and management strategies. Patient cases span across two genders and a variety of ages (ranging from 31 to 85 years old). Notably, Anti-Yo antibodies were the most frequently observed (66.7 %, 12/18 cases), followed by Anti-Hu (22.2 %, 4/18 cases), and Anti-Tr (11.1 %, 2/18 cases). Across these cases, IVIG and corticosteroids formed the cornerstone of treatment, supplemented by targeted management of the underlying malignancy.

**Table 2:** A Summary of Cases of Paraneoplastic Cerebellar Degeneration in the Literature.

**Study Author**	**Patient Age/Gender**	**Primary Tumor**	**Antibodies Identified**	**Management**
Heumer (Huemer et al., 2015)	60, Female	Poorly differentiated spindle cell carcinoma of the nasal cavity	Anti-Hu	Chemoradiotherapy
De Schamphelaere (De Schamphelaere et al., 2020)	58, Male	Unknown	Anti-Hu	N/A
Li (Li et al., 2022)	55, Female	Small cell lung cancer	Anti-Hu, Anti-Yo	IVIG, treated for SCLC outpatient
Sharobeam (Sharobeam et al., 2017)	85, Female	Grade 3 Merkel Cell Carcinoma	Anti-Hu	IV Methylprednisolone, Radiotherapy
Boldig (Boldig et al., 2024)	48, Female	Stage IV Fallopian Tube Adenocarcinoma	Anti-Yo	High Dose IV Methylprednisolone, 6 cycles of IVIG
Deac (Deac et al., 2020)	59, Female	High Grade Ovarian Serous Carcinoma	Anti-Yo	IVIG, IV corticosteroids
Martin (Martin et al., 2017)	52, Female	Stage IIIA Breast Cancer	Anti-Yo	Neoadjuvant Chemotherapy, Modified Radical Mastectomy, IVIG, Pertuzumab
Avino (Avino et al., 2023)	70, Female	Metastatic ER + Breast Cancer	Anti-Yo	2 cycles of IVIG
Jadhav (Jadhav et al., 2021)	48, Female	High Grade Breast Carcinoma	Anti-Yo	Surgery, PLEX, IVIG, Corticosteroids, Chemotherapy
Dou (Dou et al., 2024)	63, Female	Unknown	Anti-Yo	1 cycle of IVIG, Methylprednisolone, Ofatumumab
Lou (Lou et al., 2021)	61, Female	Cholangiocarcinoma	Anti-Yo	5 cycles of IVIG, IV Methylprednisolone
Yang (Yang et al., 2025)	62, Female	Squamous Cell Lung Carcinoma	Anti-Yo	Methylprednisolone, IVIG, Chemoradiotherapy
Bradley (Bradley et al., 2008)	56, Female	Stage IIIC Serous Ovarian Adenocarcinoma	Anti-Yo	Plasmapheresis, IVIG, Corticosteroids, Tacrolimus
Yan (Yan et al., 2018)	67, Female	Stage IIA Invasive Ductal Carcinoma	Anti-Yo	Left Breast Lumpectomy
Arratibel (Arratibel et al., 2020)	44, Male	Hodgkin’s Lymphoma	Anti-Tr	Chemotherapy
Kharouaa (Kharouaa et al., 2024)	61, Male	Grade 3 Merkel Cell Carcinoma	Anti-Hu	IV Methylprednisolone, Radiotherapy
Eseonu (Eseonu et al., 2010)	42, Female	Grade 3 Intraductal Breast Carcinoma	Anti-Yo	Surgical Resection, Chemotherapy, Plasma Exchange
Khan (Khan, 2018)	31, Male	Anaplastic Large Cell (Non-Hodgkin) Lymphoma	Anti-Tr	Intravenous Corticosteroids

## 4 Conclusion

Paraneoplastic cerebellar degeneration represents a rare, yet significant, manifestation of paraneoplastic neurological syndromes, emphasizing the complex interplay between malignancy and immune-mediated neuronal damage. This case is the first reported instance of Anti-Hu-associated PCD in a patient with adenocarcinoma of Müllerian origin, providing valuable insights into the limited literature. It highlights the diagnostic challenges of PCD, which necessitates a multidisciplinary approach involving physical examination, neuroimaging, cerebrospinal fluid analysis, and antibody detection. The diagnostic process is further complicated by the lack of definitive radiological findings and the potential absence of detectable antibodies in certain cases. Even with early identification and treatment, including corticosteroids, IVIG, and plasmapheresis, outcomes often remain poor, especially in cases involving Anti-Hu antibodies. This case highlights the importance of recognizing PCD in the differential diagnosis of patients with malignancy and unexplained neurological symptoms. It also emphasizes the need for continued research to refine diagnostic criteria, explore underlying disease mechanisms, and develop targeted therapies to improve patient outcomes.

Statement of Ethics: Ethical approval is not required for this study per institution guidelines. Per the IRB, a case report or limited case series is a description of the characteristics, evaluation, and/or treatment(s) of a single individual or a small group of individuals that share a common condition but did not involve activities defined as research. The retrospective review of records for publication of a single case report is not considered to be research involving human subjects; therefore, such a report does not require IRB review and approval.

## Funding Sources

The authors received no sources of funding to complete this project.

## CRediT authorship contribution statement

**Saad Rashid:** Writing – review & editing, Writing – original draft, Investigation, Data curation, Conceptualization. **Suneha Pocha:** Writing – review & editing, Writing – original draft. **Nada Saleh:** Writing – review & editing, Writing – original draft. **Erin Ozminkowski:** Writing – review & editing, Writing – original draft. **Vijayta Geeta Bansal:** Writing – review & editing, Validation, Supervision, Conceptualization. **Vibhav Bansal:** Writing – review & editing, Validation, Supervision, Conceptualization.

## Informed consent

Written informed consent was obtained from the patient for publication of this case report and accompanying images.
